# Effects of Multiple Near‐Infrared LEDs (700, 850, and 980 nm) CW‐PBM on Mitochondrial Respiration and Gene Expression in MG63 Osteoblasts

**DOI:** 10.1002/jbio.70015

**Published:** 2025-04-03

**Authors:** Simone Sleep, Deanne H. Hryciw, Laurence J. Walsh, Eliza Ranjit, Nifty Tomy, Praveen R. Arany, Roy George

**Affiliations:** ^1^ School of Medicine and Dentistry Griffith University Southport Australia; ^2^ School of Environment and Science Griffith University Nathan Australia; ^3^ Institute for Biomedicine and Glycomics Griffith University Nathan Australia; ^4^ The University of Queensland School of Dentistry Brisbane Australia; ^5^ National Centre for Disease Informatics and Research Bangalore India; ^6^ Cell Regulation and Control Unit, National Institute of Dental and Craniofacial Research Bethesda Bethesda Maryland USA

**Keywords:** gene regulation, LED, multi‐wavelength, osteogenesis, photobiomodulation, spectral width

## Abstract

**Background and Aim:**

This study evaluated mitochondrial and osteogenic activity in MG‐63 pre‐osteoblastic cells after photobiomodulation (PBM) using multiple near‐infrared LED sources (Nuralyte) emitting wavelengths from 700 to 1100 nm.

**Materials and Methods:**

MG‐63 cells were irradiated daily for 3, 5, or 7 days with energy densities of 5.3 J/cm^2^ (30 s, optimal dose) and 10.6 J/cm^2^ (60 s, high dose). Mitochondrial function was assessed using the XF Seahorse analyzer, and gene expression of osteogenic markers was analyzed.

**Results:**

Maximal mitochondrial oxygen consumption rate (OCR) significantly decreased at the optimal dose but increased at the high dose (*p* < 0.001) in 5‐day irradiated cultures. Upregulation of osteogenic markers (OCN, OPN, BMP‐2, COL‐1, RUNX2) occurred after 3–5 consecutive days of irradiation, with greater activation at the optimal dose.

**Conclusion:**

MG‐63 cells respond to PBM using MNI‐LEDs (700, 850, 980 nm) by modulating mitochondrial respiration and boosting bone‐related gene expression in a dose‐ and time‐dependent manner.

## Graphical abstract

A multi‐wavelength near infrared LED light source (700–1100 nm) altered mitochondrial respiration and energy production in MG‐63 osteoblastic cells. This LED light source boosted gene expression of the osteogenic markers ALP, RUNX2, BMP, COL1, OPN, and OCN.

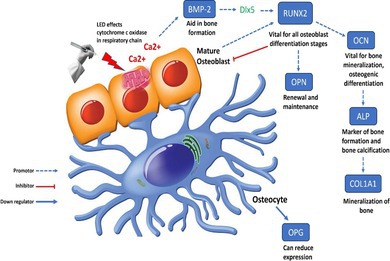

## 1 Introduction

Light sources such as lasers and light emitting diodes (LEDs) that emit light in the visible red or near infrared regions have been used for photobiomodulation (PBM). Such light sources have been used to stimulate different biological processes, such as angiogenesis and osteogenic differentiation, and to enhance wound healing and tissue regeneration [1, 2].

Single‐wavelength lasers are used currently in clinical applications to enhance cellular repair, reduce inflammation, or boost regenerative processes [3]. Mechanistically, in single‐wavelength PBM, the emitted light interacts with chromophores in cells, such as cytochrome c oxidase (CCO) in the mitochondria. This triggers further downstream effects, such as changes in the levels of reactive oxygen species (ROS), nitric oxide (NO), and ATP [4, 5, 6]. PBM can also exert effects that are mediated outside the chromophore photoacceptor of the pathway CCO–NO–ATP axis, including modulating gene expressions via other modes of actions [7].

A multi‐wavelength light source, with its broader range of absorption, can influence cells through various pathways, including water absorption, which affects cellular hydration and ion channels, in addition to the traditional cytochrome c oxidase (CCO) pathway. In contrast, single‐wavelength PBM operates within a narrower absorption spectrum determined by the specific wavelength used, typically targeting specific chromophores like cytochrome c oxidase (CCO). Single‐wavelength PBM primarily enhances cellular functions by boosting ATP production, nitric oxide (NO) release, and reactive oxygen species (ROS) levels via CCO activation, which is well documented in studies [8].

Recent studies suggest that the use of multiple wavelengths of light for PBM therapy may offer enhanced benefits for bone healing and regeneration, when compared to using single wavelengths of light [2, 9]. Using multiple LEDs can significantly boost the expression of key genes involved in bone formation, such as alkaline phosphatase (ALP), runt‐related transcription factor 2 (Runx2), bone morphogenetic proteins (BMP), type I collagen (COL1), osteopontin (OPN), and osteocalcin (OCN), in cell culture model systems [9]. Recently, a patented device with a proprietary blend of multiple near infrared light sources designed to achieve optimal PBM by activating multiple pathways was shown to be superior to single wavelength near infrared diode lasers for analgesia [10]. How this type of light source could influence bone forming cells has yet to be investigated, especially when delivered in repeated doses. Multiple doses of low‐power laser irradiation are known to be superior to single doses for boosting bone regeneration [11, 12].

To assess the influence of PBM on bone healing and regeneration, several cell culture models have proven useful. The human osteoblastic cell line MG‐63 has been used to explore osteoblast viability and differentiation, as well as mineral deposition, under a range of conditions. MG‐63 cells are stable and give consistent responses across multiple cell culture passages [13, 14]. Activation of MG‐63 cells using near infrared lasers is known to boost mitochondrial activity, leading to increased ATP production and elevated levels of ROS [2] and to enhanced expression of OCN, COL‐1, RUNX‐2, and BMP‐2 [15, 16].

The present study is the first to utilize a patented multi‐wavelength LED device to directly correlate mitochondrial oxygen consumption rates with key gene expression markers involved in osteogenesis. This approach deepens our understanding of the cellular pathways underlying tissue regeneration.

## 2 Methodology

### 2.1 Cell Culture

MG‐63 cells were obtained from the American Type Culture Collection (ATCC CRL‐1427, passage 8; Bethesda, USA) [14]. The cells were cultured in 75‐cm^2^ (*T*_75_) flasks in Dulbecco's modified Eagle's medium (DMEM, GIBCO; Invitrogen, Carlsbad, CA, USA) supplemented with 10% fetal calf serum and 1% penicillin–streptomycin solution at 37°C in a humidified atmosphere of 5% CO_2_. At confluence, cells were detached using 0.05% trypsin/EDTA solution and then plated into Seahorse microplate 8‐well plates at a density of 8 × 10^3^ cells/well. For all treatments, wells that were protected fully from exposure to near infrared light served as controls.

### 2.2 LED Treatment Schedule

The treatment schedule involved exposing MG‐63 cells to defined parameters of light, designed to be around the optimal range for stimulation using PBM (~ 4–6 J/cm^2^), and at twice that level. The LED light source (Nuralyte, Dentroid, Canberra, Australia) emitted near infrared light from 700 to 1100 nm in continuous wave mode in the same spatial orientation with a Gaussian beam profile. As shown in Table 1, the peak emissions 700, 850, and 980 nm had a beam power of 40%, 45%, and 15% respectively. The LED device was securely positioned using a clamp stand, allowing the terminal end of the light delivery system to remain perpendicular and flush on the top surface of each well, with a spot size of 7 mm diameter (area 0.39 cm^2^). The laser tip was positioned approximately 1 mm above the cell culture, with a divergence angle of 70.9° (35.45°× 2) in air and a corrected divergence angle of 53.4°(26.7°× 2) in the DMEM medium (approx. refractive index of 1.33), where refraction reduces the beam's spread compared to air. A Gaussian beam emitted from a 7 mm diameter tip has its maximum intensity at the center, decreasing toward the periphery. The full width at half maximum (FWHM) for this beam is approximately 2.98–3.2 mm in diameter. This indicates that a significant portion of the beam, specifically a 3.81 mm diameter region at the bottom of the well, irradiates the monolayer cells with the appropriate dosage of light. The optical emissions from the delivery system were measured using a laser power meter with an external sensor (Nova II, Ophir Optronics Solutions Ltd., Jerusalem, Israel) with the laser power meter set to different nominal wavelengths across the emitted range (Table 1).

**TABLE 1:** LED parameters.

Wavelength	Energy density (30 s)	Energy density (60 s)	Power (mW)	Spectral width	Beam power
700 nm	6.07 J/m^2^	12.14 J/m^2^	202.4 mW	40 nm	40%
850 nm	5.62 J/m^2^	11.24 J/m^2^	187.3 mW	50 nm	45%
980 nm	8.02 J/m^2^	16.04 J/m^2^	267.3 mW	80 nm	15%
Average	6.57 J/m^2^	13.14 J/m^2^	219.0 mW		

*Note:* All wavelengths are emitted together at the same time in CW mode, and all are in the same spatial orientation with a Gaussian beam profile.

**FIGURE 1:**
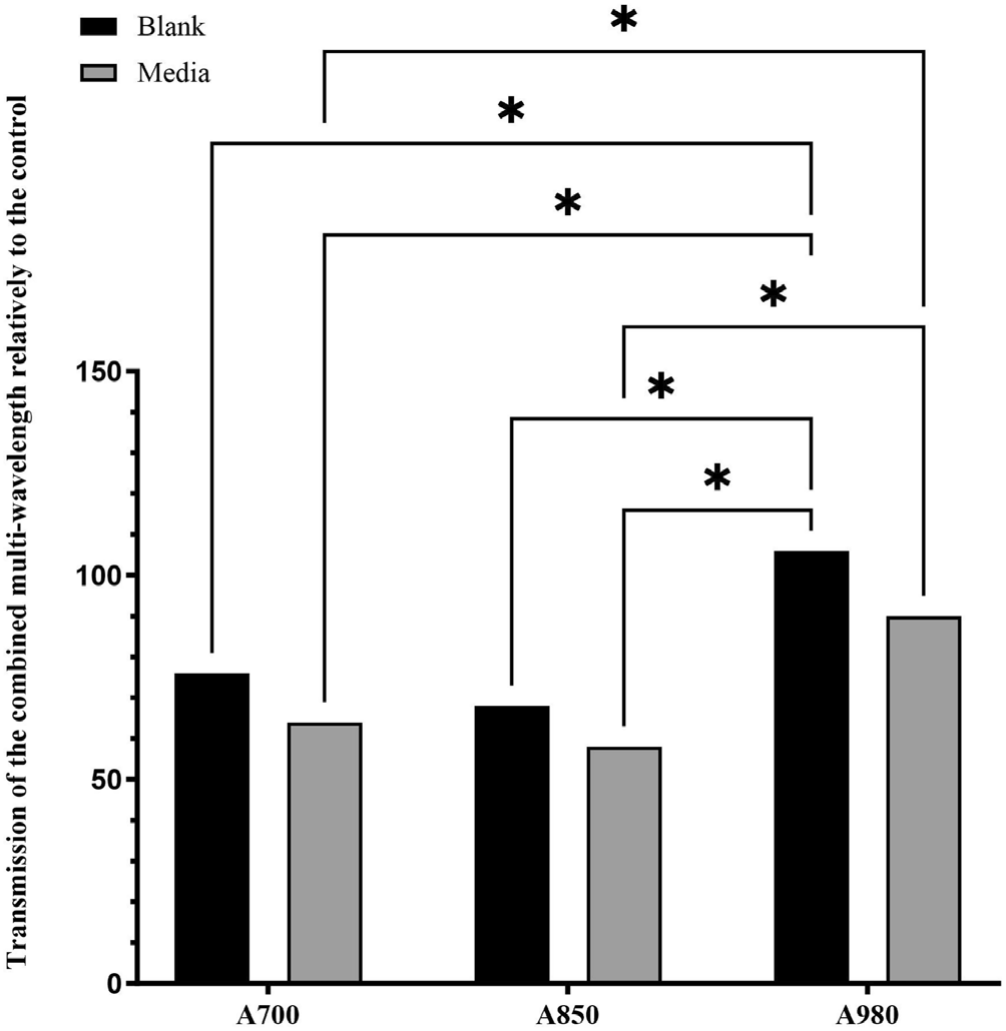
Relative energy transmission through empty tissue culture plates (blank) and DMEM culture medium at different wavelengths. Significant differences * = *p* < 0.01, *n* = 1.

The actual emitted power for the device at the level of the cell monolayer growing in the culture plate was determined using the same laser power meter. This involved placing the sensor of the power meter below the culture plate and comparing the delivered power from the LED through the culture plate, with and without DMEM culture medium in the wells. The power attenuation caused by the culture plate and the culture medium was calculated as a percentage value for each wavelength, relative to the control value at the end of the delivery system (Figure 1).

Based on past cell culture studies using diode lasers [9], it was expected that the optimal energy density for LED would be 4–6 J/cm^2^. When the attenuation of the culture plate and culture medium were both factored in (an average of 19%), the estimated energy density at the level of the cell monolayer for the optimal LED setting (30 s exposure time) was 5.3 J/cm^2^, which was within this pre‐determined optimal range. The area of monolayer cultured cells irradiation was 11.40 mm^2^.

The LED treatment schedule is shown in Table 2. Cells were treated daily for 3 days (Group A), for 5 days (Group B) or for 7 days (Group C), using exposure times of 30 or 60 s. This corresponded to estimated energy densities of 5.3 J/cm^2^ and 10.6 J/cm^2^ at the level of the cell monolayer, and these will subsequently be referred to as “optimal dose LED” and “high dose LED”, respectively. Group C was split into two subgroups so that the effects of the final LED treatment on mitochondrial respiration could be evaluated before Seahorse analysis either immediately (subgroup C.1; 0‐min) or after 24 h (subgroup C.1; 24‐h). There were 4 replicate plates for each experimental series.

**TABLE 2:** PBM treatment schedule.

Group	Treatment duration	Subgroup	Timing before seahorse analysis
A	3 days	30 s and 60 s	n/a
B	5 days	30 s and 60 s	n/a
C1	7 days	30 s and 60 s	Immediately post treatment
C2	7 days	30 s and 60 s	24 h post treatment

### 2.3 Cellular Bioenergetics

**FIGURE 2:**
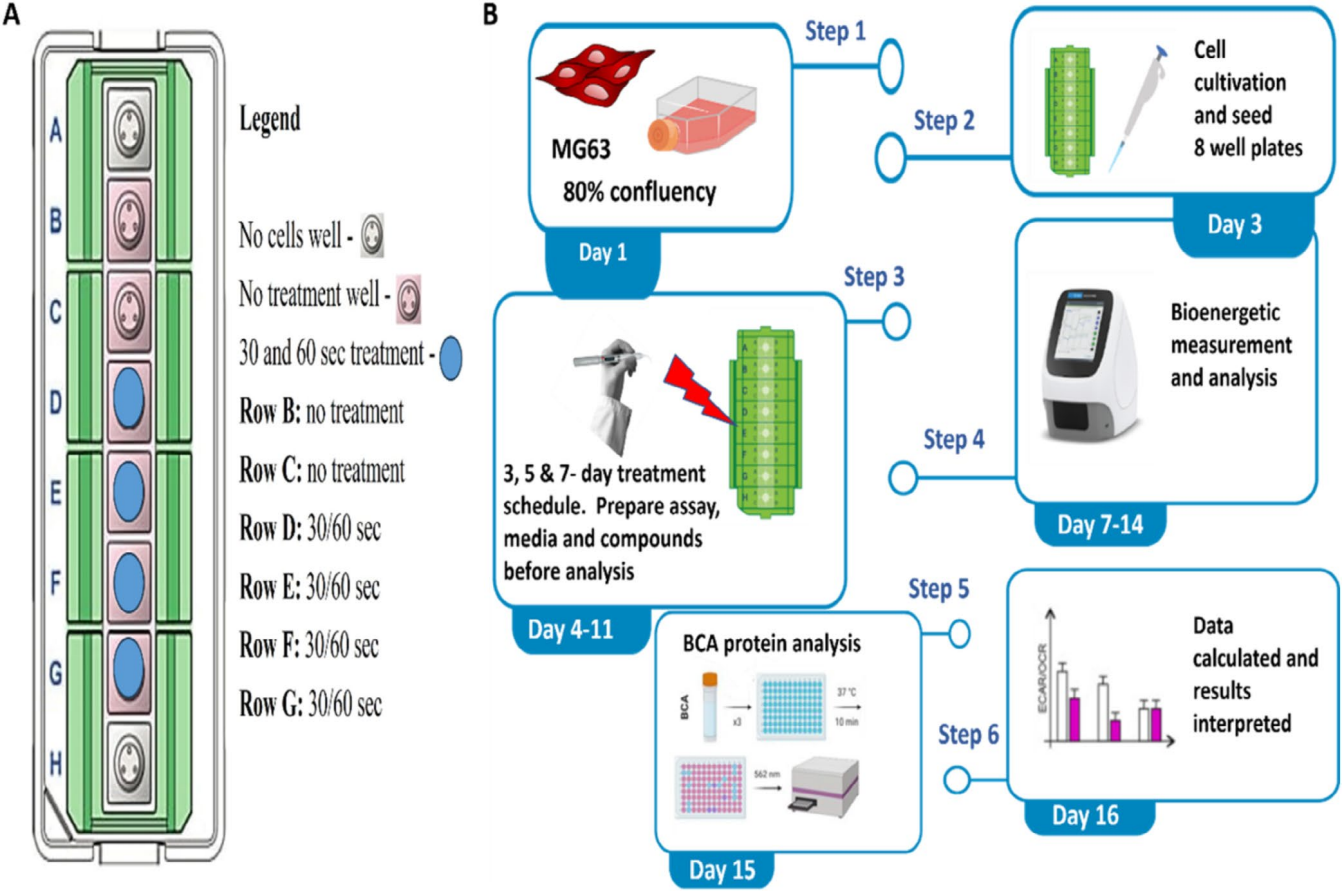
Schematic diagram of plate design and experimental flow for light irradiation experiments. (A) XF Seahorse plate design showing well design for different irradiation protocols. (B) Real‐time extracellular flux analysis for living cells using the MG‐63 cell line showing the flow of experiments.

Changes in mitochondrial function were assessed using the XF Seahorse analyzer (Agilent Technologies, Santa Clara, CA, USA) to measure mitochondrial respiration. MG‐63 cells were plated in 8‐well tissue culture‐treated Seahorse extracellular flux (XF) 8 microplates (Agilent Technologies, Santa Clara, CA, USA) at a density of 2–5 × 10^3^ cells per well. The experimental workflow is summarized in Figure 2.

Cells were incubated for 1 day to allow adhesion. The XF Sensor Cartridge was hydrated overnight using ultrapure water, then incubated with XF Calibrant solution at 37°C in a CO_2_‐free, humidified incubator for 60 min. Cells were washed and incubated with XF assay medium, pH 7.4 at 37°C. This was made by adding 10 mM D‐glucose, 1 mM sodium pyruvate, and 200 mM glutamine solution (Seahorse XF #103579‐100, Agilent Technologies, USA), according to the manufacturer's recommended protocol. This assay medium ensures that the cells are in an environment that supports their normal metabolic functions.

Cells were exposed to Seahorse Mito Stress assay medium (Agilent #103325‐100) and then mitochondrial respiration was assessed using the oxygen consumption rate (OCR), and glycolysis was assessed using the extracellular acidification rate (ECAR). Both mitochondrial respiration and glycolysis are crucial for energy production. Data for OCR were adjusted based on protein concentration and then used to compute multiple mitochondrial metrics using previously validated techniques [17].

### 2.4 BCA Protein Normalization

Protein concentrations were assessed in triplicate utilizing the bicinchoninic acid method (Pierce BCA Protein Assay Kit, Thermo Fisher Scientific #23227, USA) following the manufacturer's instructions, using bovine serum albumin as a control.

### 2.5 Osteogenic Gene Expression

Gene expression in MG‐63 cells at 3 days was assessed using real‐time PCR. Total RNA was collected from MG‐63 cells (10 000 cells per disc) (Qiagen RNeasy Mini Kit, Qiagen GmbH, Germany) and its purity checked (ND 1000, NanoDrop, Biolab, Australia) before being transcribed into complementary DNA (QuantiTect Reverse Transcription Kit, Qiagen, Germany). Gene expression of osteocalcin (OCN), osteopontin (OPN), alkaline phosphatase (ALP), runt‐related transcription factor 2 (RUNX2), collagen type I (COL‐1), and bone morphogenetic protein‐2 (BMP‐2) was determined using the real‐time quantitative polymerase chain reaction (qPCR) (QuantStudioTM 6 Flex, Thermo Fisher Scientific, USA), employing the SensiFAST SYBR No‐ROX kit (Bioline Aust Pty Ltd., Australia) with the following thermal cycling conditions: initial denaturation at 95°C for 2 min then 40 cycles of denaturation at 95°C for 5 s, and extension at 60°C for 30 s. Osteogenic gene primer sequences [18] are listed in Table 3. All reactions were run in triplicate. The *C*_*t*_ values of osteogenic genes were normalized using the housekeeping gene glyceraldehyde 3‐phosphate dehydrogenase (GAPDH).

**TABLE 3:** Primer sequences for osteogenic genes used for qPCR.

Gene	Forward primer (5′–3′)	Reverse primer (5′–3′)
GAPDH	CGCTCTCTGCTCCTCCTGTT	CCATGGTGTCTGAGCGATGT
OCN	ATGAGAGCCCTCACACTCCTC	GCCATAGATGCGTTTGTAGGCG
OPN	CTCCATTGACTCGAACGACTC	GACACAGCATGAAAGGAGAAAG
BMP‐2	CCAAGTAAGTCCAACGAAAG	TCTGTTTGCGACTTCAGTGGTA
RUNX2	ATGGCGGGTAACGATGAAAAT	ACGGCGGGGAAGACTGTGC
COL‐1	AGGGCCAAGACGAAGACATC	AGATCACGTCATCGCACAACA
ALP	ACAAGCACTCCCACTTCATC	CCATGGTGTCTGAGCGATGT

### 2.6 Statistical Analysis

Data analyzes were carried out using Prism software version 8.4.1 (GraphPad, San Diego, CA, USA). Statistical analyzes were performed to assess differences in mitochondrial metabolism between the treatment groups. Because the sample sizes varied, the data sets were normalized, with input from a statistician. Parametric tests (*t*‐tests, ANOVA) and non‐parametric tests (Kruskal–Wallis, Mann–Whitney *U*) were used to compare mitochondrial activity between PBM‐treated cells and control groups. Each experiment was repeated at least twice to ensure reproducibility.

## 3 Results

### 3.1 Seahorse XF Mitochondrial Respiration

**FIGURE 3:**
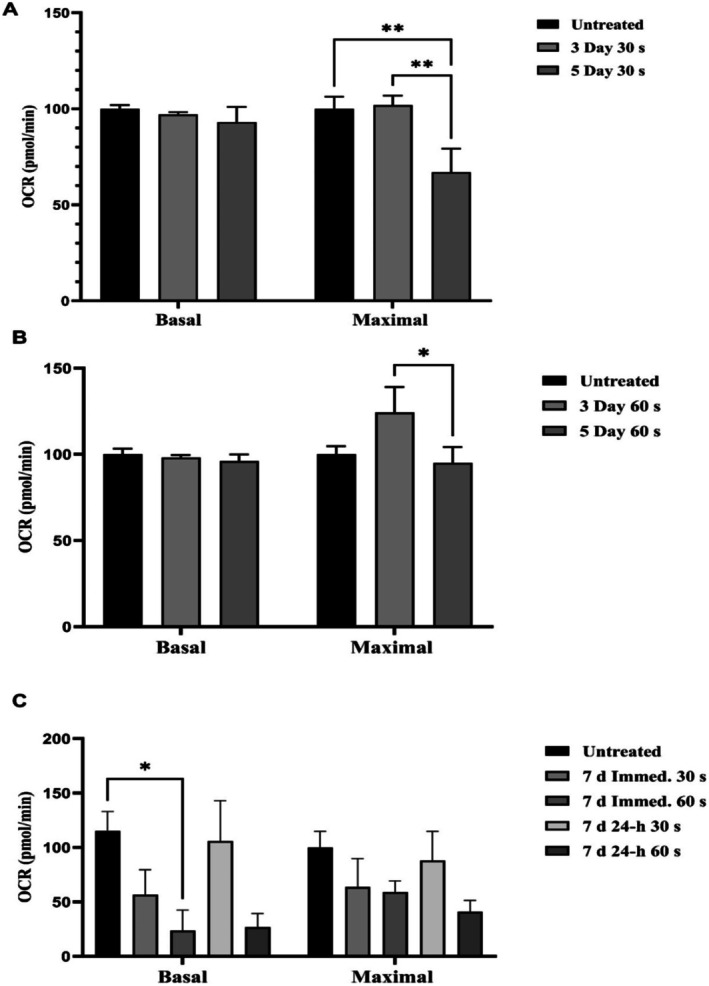
Mitochondrial respiration changes with PBM. Panel A shows significant increase of maximal respiration on the 3‐day, and significant decrease on the 5‐day cultures treated with PBM for 30 s compared to untreated, while panel B shows a significant increase of maximal respiration at 3‐day at 60 s post PBM. Panel C shows 7‐day cultures assessed immediately or 24 h after PBM with a significant decrease of basal respiration at 7‐day immediate at 60 s. * = *p* < 0.01, ** = *p* < 0.001. *n* = 8.

For a 30 s dose PBM (Figure 3A), in the 3‐day cultures (group A), there were no significant differences in either basal OCR or maximal OCR between untreated and treated samples. In contrast, in the 5‐day cultures (group B), there was a significant reduction in maximal OCR compared to both untreated cells and to 3‐day cultures (*p* < 0.001), but no change in basal OCR. For a 60 s dose PBM (Figure 3B), maximal respiration was significantly increased in the 5‐day cultures, while basal respiration was unaffected. In 7‐day cultures (Figure 3C), PBM significantly suppressed basal respiration (*p* < 0.01), and there was a trend for maximal respiration to also be suppressed, but this did not reach the threshold for statistical significance. Overall, these results show that the dose and the timing of irradiation both influence what changes occur in mitochondrial respiration.

### 3.2 Osteogenic Gene Expression After PBM

**FIGURE 4:**
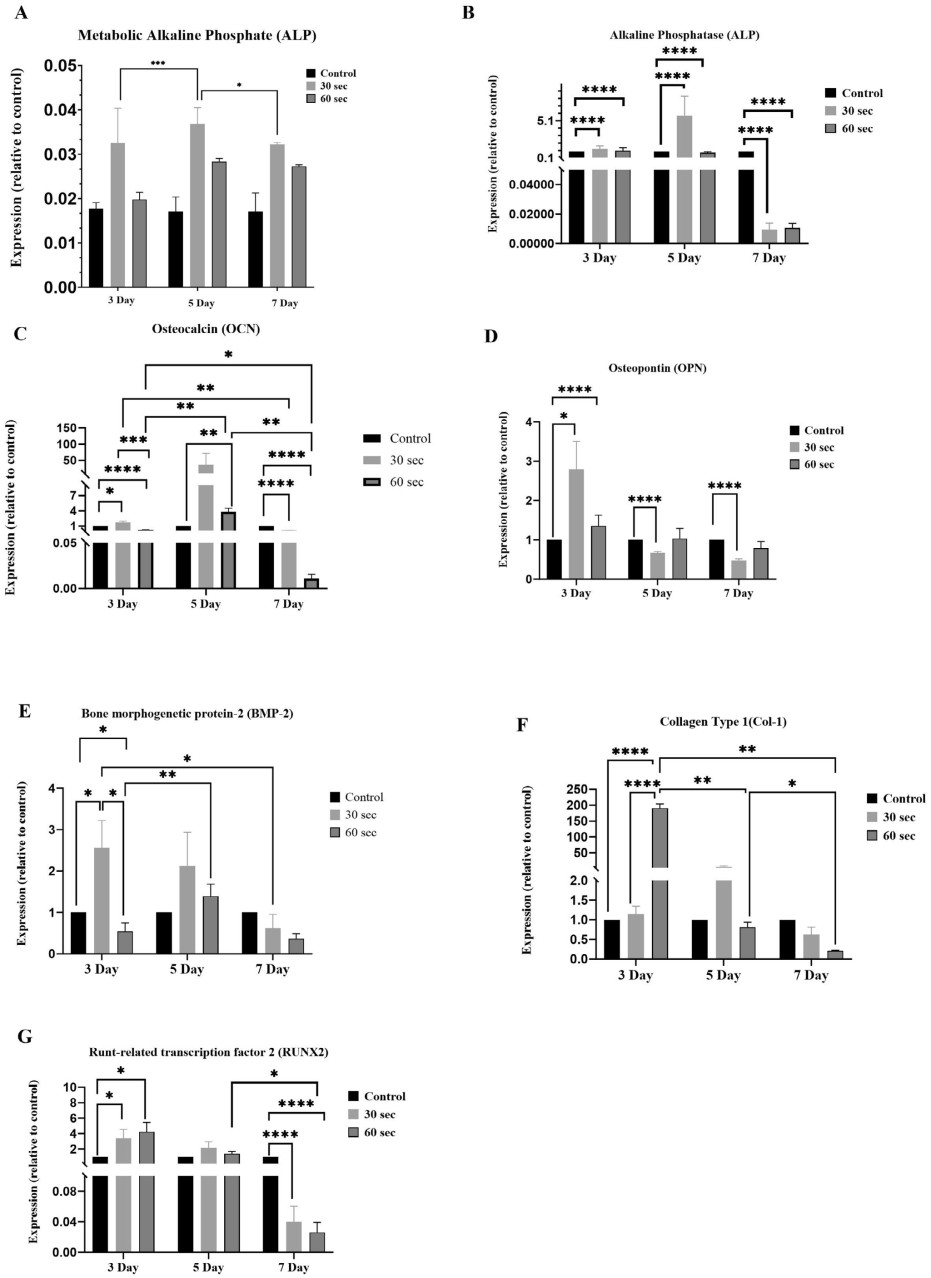
Gene expression of MG63 cells at 3, 5, and 7 days of PBM. Data show means and standard deviations. (A) Metabolic ALP increased significantly at 5 and 7 days, (B) ALP expression peaked at 5 days with significant increases across all conditions, (C) OCN highest expression at 7 days, with significant differences between groups, (D) OPN significantly increased at 3 days and reduced expression at later time points, (E) BMP‐2 increased significantly at 3 and 5 days, (F) Col‐1 increased at 3 days, declining thereafter, (G) RUNX2 significantly increased at 3 and 5 days and significant differences across conditions. Asterisks indicate statistically significant changes *p < 0.01, **p < 0.001, ***p < 0.0001, ****p < 0.00001. There were three replicates in each experimental series.

Data for the expression of genes that are critical in osteogenesis are presented in Figure 4. Overall, PBM at the optimal dose was far more effective at enhancing gene expression than high‐dose PBM.

Metabolic ALP activity was enhanced by optimal dose PBM in 5‐day cultures compared to days 3 and 7 (Figure 4A), and this corresponded with significantly increased expression of the ALP gene, which also rose at Day 3 and peaked on Day 5 (Figure 4B). With high dose PBM, the enhancement of ALP gene expression was less prominent than for optimal dose PBM. ALP gene expression declined significantly in 7‐day cultures, compared to untreated controls, for both PBM doses.

A similar pattern of results was seen for the osteocalcin gene (Figure 4C), which rose in Day‐3 cultures compared to the control, peaked at Day 5, and then declined at Day 7. Once again, stronger effects were seen for optimal dose PBM than for high dose PBM.

For osteopontin, gene expression was enhanced in 3‐day cultures for both PBM doses, with the optimal dose being the most effective stimulus at this time point. OPN expression then declined at 5 and 7 days (Figure 4D).

Changes in BMP‐2 expression with optimal dose PBM followed the same pattern as OPN, with a peak at Day 3 followed by a subsequent decline (Figure 4E). Conversely, high dose PBM caused increased BMP‐2 gene expression on Day 5.

For type 1 collagen, there was greater stimulation of Col‐1 expression for high dose PBM than for low dose PBM (Figure 4F). The former caused Col‐1 gene expression to peak at Day 3, while the latter caused a smaller peak at Day 3.

For RUNX2, both PBM doses caused a significant increase at Day 3, after which levels declined (Figure 4F). There was no notable difference between the two PBM doses at any of the three time points.

Overall, these results show dose‐related effects, with Col‐1 responding more to high‐dose PBM, with other markers more responsive to optimal‐dose PBM, and reaching their peak earlier at Day 3 rather than later at Day 5. Low expression of osteogenic markers in 7‐day cultures was a consistent finding with high‐dose PBM, consistent with a suppressive effect.

## 4 Discussion

The results of the current study show that the near infrared light from 700 to 1100 nm emitted from an LED device can alter the behavior of MG‐63 osteoblastic cells, with altered mitochondrial respiration and changes in osteogenic gene expression. Both were influenced by the PBM dose used and the treatment regimen.

PBM affected maximal respiration more noticeably than basal respiration, with a time‐ and dose‐dependent response. In 3‐day cultures, the pre‐determined optimal PBM dose increased maximal respiration. Prolonged daily treatments at optimal and high PBM doses suppressed maximal respiration (i.e., 5 and 7 days). The response pattern also suggests that the cells are initially stimulated over the first 3 days, after which basal respiration declines, as a feedback response.

PBM treatment at both optimal (30 s) and high doses (60 s) exerted a time‐dependent effect on the expression of genes related to bone remodeling in MG‐63 cells. Expression of the ALP gene correlated with elevated ALP activity. A simultaneous increase in ALP gene expression and enzyme activity suggests the cells were transitioning from early to later stages of osteoblast differentiation.

PBM caused elevations in OPN, BMP‐2, OCN, RUNX2, and Col‐1 gene expression, with some markers being elevated at 3 days and others reaching their peak at 5 days, according to the PBM dose used. A difference in the activation sequence of genes is not unexpected, given the chronology of mineral deposition, where matrix formation is followed by mineral deposition. The finding that PBM with near infrared LED light can stimulate both the early and late stages of osteogenesis makes this approach a potential tool for promoting bone healing and regeneration.

The current findings reveal both similarities and differences when compared to previous studies that employed diode lasers for PBM treatments on MG‐63 cells [19]. Additionally, the impact of PBM, delivered through multi‐wavelength lasers and LEDs, on mitochondrial respiration and the expression of osteogenic markers has been extensively documented in the literature [16, 19]. Exploring novel wavelength combinations and dose–response relationships facilitates a deeper understanding of the influence of wavelength, along with the corresponding effects of dose‐related parameters and treatment duration.

A study using an 808 nm laser showed enhanced mineral formation, but no effect on osteogenic differentiation, based on ALP activity and gene expression results [20]. Both ALP and COL‐1 gene expression were reduced at 7 days. The energy densities used were, however, much lower than those employed in the present study (0.5 and 1 J/cm^2^) [20]. The effects of PBM on osteogenesis depend on several factors, with energy density being the most crucial, provided the wavelength is appropriate [21]. Depending on the settings that are used, PBM using near‐infrared diode lasers has been shown to significantly boost MG‐63 expression of OCN, COL‐1, RUNX‐2, and BMP‐2 [21]. The present results extend these earlier findings by demonstrating elevated expression of these markers, as well as ALP and OPN, using an LED light source.

The observation that OPN is enhanced by PBM and then declines is consistent with the role of OPN in early bone remodeling processes. Using PBM to boost OPN gene expression may have relevance to stimulating the formation of new bone, for example for repairing bone defects [22]. The fact that increased expression of BMP‐2 also was seen after PBM is important, since this marker corresponds to enhanced bone formation. Past studies have shown PBM using diode lasers can stimulate the expression of BMP‐2 in a highly dose‐dependent manner [23].

Stimulatory effects of PBM on collagen synthesis by fibroblasts occur when PBM is delivered using a diode laser daily for three consecutive day [24]. This aligns with the present study that shows that the LED device can enhance COL‐1 expression when applied on target cells for 3 consecutive 3 days.

In the present study, PBM caused an increase in RUNX2 expression on Day 5. This key transcription factor is necessary for osteoblast differentiation, which in turn is essential for the initiation of bone formation [25]. Outside of the setting of bone regeneration, the synthesis of new extracellular matrix components by fibroblasts can also be stimulated by PBM^26^, with lowered expression of matrix metalloproteinase enzymes and reduced matrix degradation, as well as greater synthesis of new matrix components [26]. The present findings support the notion that there is an LED PBM effect on both the early and late stages of osteogenesis. PBM‐LED enhances osteoblast proliferation and maturation by stimulating mitochondrial bioenergetics, increasing ATP production, and activating key signaling pathways such as TGF‐β, BMP, and MAPK, which collectively promote osteogenic differentiation and bone matrix formation [27]. Further studies are needed to assess how PBM delivered using the LED device influences fibroblasts.

The present results for mitochondrial respiration and osteogenic gene expression are broadly consistent with the expectation that PBM doses from 4 to 6 J/cm^2^ would stimulate normal cellular functions. Multi‐wavelength LED PBM may boost mitochondrial respiration by activating CCO, increasing ATP and ROS signaling, which triggers redox‐sensitive transcription factors (NRF2, NF‐κB) to upregulate osteogenic genes (RUNX2, ALP, COL1A1), promoting bone formation and differentiation [5].

The present study considered the dose received by MG‐63 cells when absorption in the culture plates and culture medium had been factored in when determining what the optimal PBM dose would be, and the doubled that for the high PBM dose. The high dose PBM treatment gave more pronounced declines in both respiration and gene expression in the day‐7 cultures. This is in line with the Arndt‐Schultz dose–response curve for PBM using near infrared laser light sources [28, 29], were using doses well above the optimal activation level gives suppressive actions.

The present study used an LED light source that emitted light from 700 to 1100 nm with three peaks. The lower parts of this wavelength range fall within the known absorption spectrum for CCO, and this explains why it can cause effects similar to those seen for diode laser wavelengths in the same wavelength range [30]. Choosing different light wavelengths will alter the response of cells and tissues to PBM, according to the activation of chromophores that the light induces [5, 28]. Because of its broad spectral range, the LED light source used in the present study should be able to activate multiple pathways at once, leading to greater effects on cells than using a single laser wavelength that can activate fewer chromophores.

A study by Hong (2018) showed pulsed wave PBM enhances osteogenesis more effectively than continuous wave by preventing heat buildup, optimizing ATP production, and boosting bone‐forming gene activation [31]. The present study used continuous wave; however, with a beam power of less than 50% for each peak wavelength. Further recent work using the same multi‐wavelength LED light source in a clinical trial reported that no discomfort occurred from photothermal effects, unlike the case for pulsed 904 nm and continuous wave 808 nm lasers under comparable irradiation conditions when used on healthy adult human premolar teeth [10].

Using a monolayer cell‐line model in this study provides a controlled environment for investigating PBM effects on osteoblast mitochondrial activity; however, it lacks the complex extracellular matrix interactions, biomechanical loading, and nutrient gradients present in 3D bone models or bioreactor‐based studies, which would offer a more physiologically relevant understanding of bone regeneration. Further studies are needed to examine how the activation pattern of the LED light source compares to single‐wavelength monochromatic light sources such as diode lasers of various types, in terms of effects on cell growth, differentiation, and gene expression. This will be relevant to how different PBM light sources are used to promote healing in different clinical situations.

## 5 Conclusions

MG‐63 cells respond to PBM with MNI‐LEDs (700, 850, and 980 nm) continuous wave source: modulating mitochondrial respiration and boosting the expression of genes related to bone formation in a dose‐dependent and timing of irradiation manner.

## Author Contributions

**Simone Sleep, Roy George:** conceptualization. **Simone Sleep, Roy George, Eliza Ranjit and Deanne H. Hryciw:** methodology. **Simone Sleep, Roy George, Eliza Ranjit and Deanne H. Hryciw:** validation. **Nifty Tomy:** formal analysis. **Simone Sleep, Roy George, Eliza Ranjit:** investigation. **Simone Sleep, Roy George:** resources. **Simone Sleep, Roy George, Eliza Ranjit, Laurence J. Walsh, Praveen R. Arany:** data curation. **Simone Sleep:** writing – original draft preparation. **Simone Sleep, Roy George, Eliza Ranjit, Nifty Tomy, Laurence J. Walsh, Praveen R. Arany and Deanne H. Hryciw:** writing – review and editing. **Roy Georg:** principal Supervisor. **Deanne H. Hryciw, Praveen R. Arany:** associate supervision.

## Conflicts of Interest

Laurence Walsh is the named inventor of the Nuralyte LED device used in this study. The other authors declare no conflicts of interest.

## Data Availability

The data that support the findings of this study are available from the corresponding author upon reasonable request.
